# Comparative expression pattern of Matrix-Metalloproteinases in human glioblastoma cell-lines and primary cultures

**DOI:** 10.1186/1756-0500-3-293

**Published:** 2010-11-10

**Authors:** Carsten Hagemann, Jelena Anacker, Stefanie Haas, Daniela Riesner, Beate Schömig, Ralf-Ingo Ernestus, Giles H Vince

**Affiliations:** 1 University of Würzburg, Department of Neurosurgery, Tumorbiology Laboratory, Würzburg, Germany; 2 Department of Gynaecology and Obstetrics, University of Würzburg, Würzburg, Germany; 3 Department of Internal Medicine I, Laboratory of Experimental Rheumatology and Neuroendocrino-Immunology, University of Regensburg, Regensburg, Germany

## Abstract

**Background:**

Glioblastomas (GBM), the most frequent malignant brain tumors in adults, are characterized by an aggressive local growth pattern and highly invasive tumor cells. This invasion is facilitated by expression of matrix metalloproteinases (MMPs), a family of zinc-dependent endopeptidases. They mediate the degradation of protein components of the extracellular matrix. Twenty-three family members are known. Elevated levels of several of them have been reported in GBM. GBM cell-lines are used for *in vitro *studies of cell migration and invasion. Therefore, it is essential to know their MMP expression patterns. Only limited data for some of the cell-lines are published, yet. To fill the gaps in our knowledge would help to choose suitable model systems for analysis of regulation and function of MMPs during GBM tumorigenesis, cell migration and invasion.

**Findings:**

We analysed MMP-1, -8, -9, -10, -11, -13, -17, -19, -20, -21, -23, -24, -26, -27, and MMP-28 expression in seven GBM cell-lines (SNB-19, GaMG, U251, U87, U373, U343, U138) and in four primary cell cultures by semiquantitative RT-PCR, followed changes in the MMP expression pattern with increasing passages of cell culture and examined the influence of TNF-α and TGF-β1 stimulation on the expression of selected MMPs in U251 and U373 cells.

MMP-13, -17, -19 and -24 were expressed by all analyzed cell-lines, whereas MMP-20 and MMP-21 were not expressed by any of them. The other MMPs showed variable expression, which was dependent on passage number. Primary cells displayed a similar MMP-expression pattern as the cell-lines. In U251 and U373 cells expression of MMP-9 and MMP-19 was stimulated by TNF-α. MMP-1 mRNA expression was significantly increased in U373 cells, but not in U251 cells by this cytokine. Whereas TGF-β1 had no impact on MMP expression in U251 cells, it significantly induced MMP-11 and MMP-24 expression in U373 cells.

**Conclusions:**

Literature-data and our own results suggest that the expression pattern of MMPs is highly variable, dependent on the cell-line and the cell-culture conditions used and that also regulation of MMP expression by cytokines is cell-line dependent. This is of high impact for the transfer of cell-culture experiments to clinical implementation.

## Findings

### Background

Glioblastomas (GBM) are the most common malignant brain tumors in adults [1]. Patients have very limited prognosis due to the aggressive local growth pattern of these tumors [2-4]. Invasion of tumor cells into the healthy brain tissue is facilitated by expression of different proteolytic enzymes like matrix metalloproteinases (MMPs), a family of zinc-dependent endopeptidases [5,6]. They mediate the degradation of protein components of the extracellular matrix [7]. To date, 23 members of the human MMP gene family are known [8]. Elevated levels of several MMPs, like for example MMP-1, -2, -7, -9, -11, -12, -14, -15, -19, -24 and -25 have been reported in malignant glioma samples from patients [6,9-20], suggesting that their expression is closely related to malignant progression *in vivo*.

Human GBM cell-lines are used for *in vitro *studies of cell migration and invasion [21-24] and numerous studies investigated expression of selected MMPs in human GBM cell-lines (Table 1) [10,15,19,20,25-36]. However, several MMPs have not been analysed in these cells, yet. Therefore, there are large gaps in our knowledge about MMP expression in human GBM cell-lines. To fill these gaps would help to choose suitable model systems for the analysis of regulation and function of MMPs during GBM tumorigenesis, cell migration and invasion.

**Table 1:** MMP expression in glioblastoma cell-lines

	SNB-19	GaMG	U251	U87	U373	U343	U138
**MMP-1**	**+**	**+**	**+**^25,31,35^	**+**/-^15,28,35^	**+**^35^	**+**^25^	**+**
**MMP-2**	+^19,20^	+^19^	+^19,20,25,26,31,35,36^/-^33^	+^10,15,28,33,35,36^	+^10,19,35,36^	-^25^	+^19,36^
**MMP-3**	+^19^	-^19^	+^19,31,6^	+^15,36^/-^28^	+^36^/-^19^		+^19^/-^36^
**MMP-7**	+^19^	-^19^	+^19,26,31,35,36^	+^15,30^/-^35^	+^19,35,36^		+^636^/-^19^
**MMP-8**	-	-	+^31^/-^26^	-	-	-	-
**MMP-9**	**+**^29,30^/-^19^	**+**^19^	**+**^19,31^/-^25,36^	**+**^10,15,36^/-^28^	**+**^10,19^/-^36^	-^25^	-^19,36^
**MMP-10**	**+**	**+**	**+**^26,31^	-	**+**	**+**	**+**
**MMP-11**	-	**+**	**+**^31^/-^35^	-^35^	+^35^/-	**+**	**+**
**MMP-12**	+^19^	-^19^	+^19,31^/-^36^	**+**^36^	-^19,36^		+^19^/-^36^
**MMP-13**	**+**	**+**	**+**^26,31^	**+**	**+**	**+**	**+**
**MMP-14**	+^20^		+^20,26,31,34-36^	+^10,15,31,32,35,36^/-^28^	+^31,35,36^/-^10^	+^31^	+^36^
**MMP-15**			+^31,34,35^	+^31,32^/-^35^	+^31^/-^35^	+^31^	
**MMP-16**			+^26,34^/-^31,35^	+^31,32,35^	-^31,35^	-^31^	
**MMP-17**	**+**	**+**	**+**^31^	**+**^31^	**+**^31^	**+**^31^	**+**
**MMP-19**	**+**	**+**	**+**^31^	**+**	**+**	**+**	**+**
**MMP-20**	-	-	-^31^	-	-	-	-
**MMP-21**	-	-	-^31^	-	-	-	-
**MMP-23**	**+**	-	**+**^31^	**+**	**+**	**+**	**+**
**MMP-24**	**+**	**+**	**+**^31,34^	**+**^31^	**+**^31^	**+**^31^	**+**
**MMP-25**			-^31^	-^31^	-^31^	-^31^	
**MMP-26**	-	-	+^31^/-^27^	-	-	-	-
**MMP-27**	-	-	+^31^/-	-	-	-	-
**MMP-28**	**+**	-	**+**^31^	-	**+**	**+**	-

+: expressed; -: not expressed; bolt: data from this study; Figures: References

In seven glioblastoma cell-lines (SNB-19, GaMG, U251, U87, U373, U343, U138) and in four primary cell cultures, established from tumor specimens analysed previously by our group [16], we performed a comprehensive study of MMP expression using semiquantitative RT-PCR. In addition, we followed changes in the MMP expression pattern with increasing passages of cell culture and we examined the influence of TNF-α and TGF-β1 stimulation on the expression of selected MMPs in U251 and U373 cells.

#### Expression of MMPs in glioblastoma cell-lines

**Figure 1:**
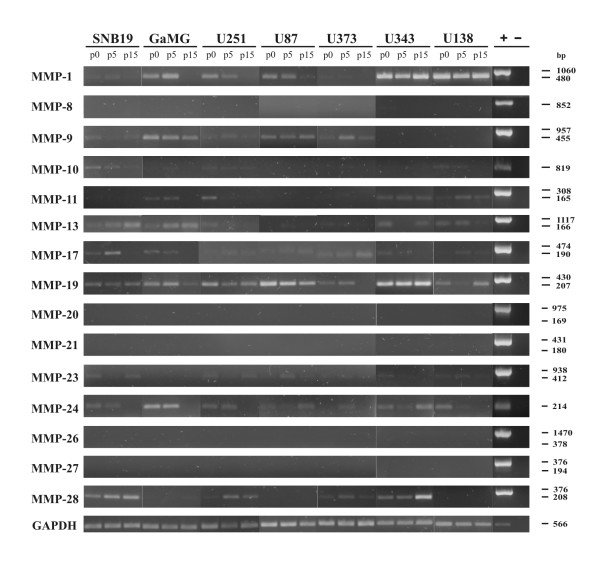
**Expression analysis of MMPs in glioblastoma cell-lines and primary cells by semiquantitative RT-PCR**. Total RNA from the seven glioblastoma cell-lines SNB-19, GaMG, U251, U87, U373, U343 and U138 was used as template for semiquantitative RT-PCR analysis. Cells were passaged fifteen times and samples of total RNA were isolated from passage 0 (p0), passage 5 (p5) and passage 15 (p15). Primers were designed in flanking exons specific for each transcript (Table 2). The length of the cDNA amplicons in base pairs (bp) is shown on the right side of the figure. As positive control (+) genomic DNA from GaMG cells was used, except for MMP-10 and MMP-24 where cDNA of HBMEK cells and for GAPDH where cDNA from U87 cells served as positive control. The various cDNA concentrations were normalized to that of the housekeeping gene GAPDH, which was used as internal loading control. Shown is one representative experiment out of six.

Expression of fifteen MMPs was analysed in the seven GBM cell-lines SNB-19, GaMG, U251, U87, U373, U343 and U138 by semiquantitative RT-PCR (Figure 1). Those MMPs were examined with no or very limited data published about their expression in GBM cell-lines. No mRNA expression was detectable for MMP-8, -20, -21, -26 and MMP-27 (Figure 1). MMP-19 mRNA was strongly expressed in all analysed cell-lines, whereas MMP-10, -17 and -23 mRNAs were only very weakly detectable (Figure 1). The other surveyed MMPs showed a diverse expression in the different GBM cell-lines (Figure 1).

**Figure 2:**
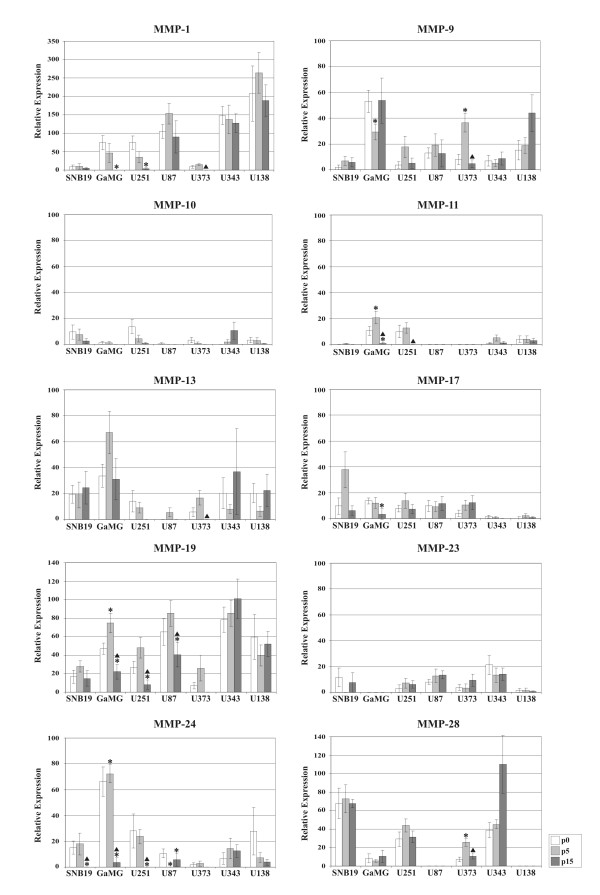
**Densitometric quantification of MMP mRNA expression in glioblastoma cell-lines**. Comparison of densitometrically quantified MMP mRNA expression by human GBM cell-lines SNB19, GaMG, U251, U87, U373, U343 and U138 in arbitrary units. Each value was normalized to the respective GAPDH mRNA expression. Shown are the means of six independent experiments. The standard errors of the mean (SE) are shown as error-bars. Asterisks indicate statistically significant alterations in expression compared to the expression in passage p0, triangles in comparison to the expression in passage p5 (two-tailed t-test, p ≤ 0.05). White columns: passage p0, light grey columns: passage p5, dark grey columns: passage p15.

These results became more obvious after densitometric quantification (Figure 2). MMP-19 was highly expressed in U87, U343 and U138 cells (Figure 1, Figure 2). Whereas its expression remained stable for 15 passages in SNB-19, U373, U343 and U138 cells, there was a statistically significant decrease of MMP-19 mRNA expression at passage 15 in GaMG, U251 and U87 cells (Figure 2). MMP-1 mRNA expression was strongest in U87, U343 and U138 cells and remained stable for 15 passages. However, it was very weakly expressed in SNB-19 and U373 cells and the intermediate expression in GaMG and U251 cells was successively reduced with passaging of cells (Figure 2). A comparable reduction of mRNA expression was found for MMP-11 in GaMG and U251 cells, MMP-17 in GaMG cells and MMP-24 in SNB-19, GaMG, U251 and U87 cells (Figure 2). MMP-10 was not detectable in U87 cells, MMP-11 not in SNB-19, U87 and U373 cells, MMP-23 not in GaMG cells and MMP-28 not in U87 cells (Figure 2). An increase of MMP expression was visible at passage 5 in U373 cells for MMP-9 and in GaMG cells for MMP-11, MMP-19 and MMP-24 (Figure 2). This increase was reduced again with further duration of culture (Figure 2). The other MMPs were expressed in varying amounts in the different cell-lines and their expression remained stable over the passages tested (Figure 2).

#### Expression of MMPs in primary glioblastoma cells

**Figure 3:**
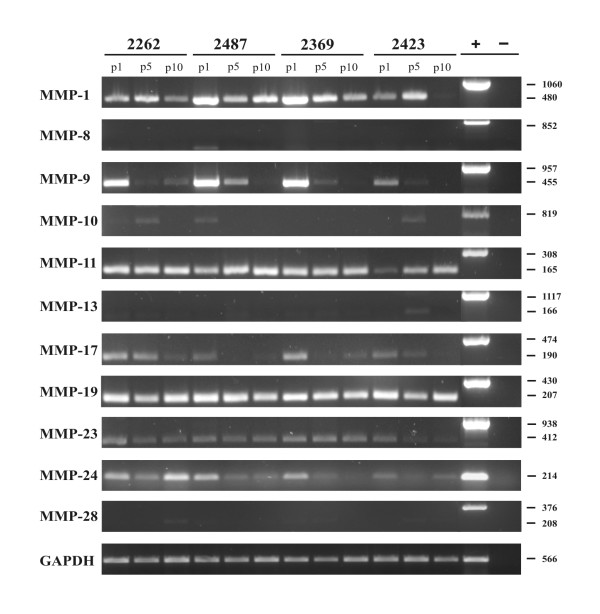
**Expression analysis of MMPs in primary glioblastoma cells by semiquantitative RT-PCR**. Four different primary cell cultures, derived from patients biopsies, were passaged ten times. Total RNA was isolated from passage 1 (p1), passage 5 (p5) and passage 10 (p10) and used for MMP specific semiquantitative RT-PCR, as described in Figure 1.

In addition to the cell-lines, four primary GBM cell cutures, which were derived from patient's tumor samples [16], were analysed for MMP-1, -8, -9, -10, -11, -13, -17, -19, -23, -24 and MMP-28 mRNA expression by semiquantitative RT-PCR (Figure 3). Very faint expression was detectable for MMP-8, -10, -13 and MMP-28 (Figure 3). Strongest expression could be found for MMP-1, -11 and MMP-19 in the four primary cells (Figure 3). MMP-17, -23 and MMP-24 showed intermediate expression (Figure 3). MMP-9 was strongly expressed in passage 1 of the primary cells, but its expression successively decreased and almost disappeared at passage 10 (Figure 3). Similar data were obtained for MMP-1, MMP-17 and MMP-24, with exception that the latter was expressed constantly in 2262 cells for the investigated 10 passages (Figure 3). Whereas MMP-23 expression remained unchanged in the primary cells 2262, 2487 and 2369, its expression was reduced with duration of cell culture in 2423 cells (Figure 3). 2423 cells also were distinctive in MMP-11 expression, since it was increased with advancing passages (Figure 3).

#### Stimulation of MMP expression by TNF-α and TGF-β1 in U251 and U373 cells

**Figure 4:**
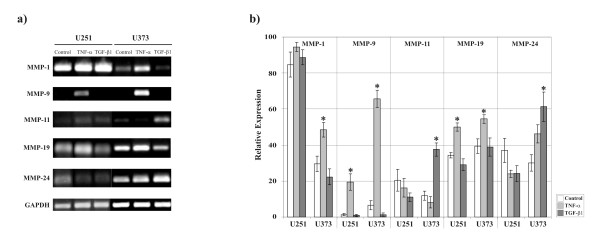
**Regulation of MMP mRNA expression by TNF-α and TGF-β1**. **a) **U251 and U373 cells were successively adjusted to grow in medium without FCS and stimulated with 10 ng/ml TNF-α and TGF-β1 for 48 h, respectively. Control cells were treated with PBS/BSA solution instead. Total RNA was isolated from these cells and MMP expression strength analysed as explained in Figure 1. Shown is one representative experiment out of six. **b) **Densitometric quantification of MMP mRNA regulation by TNF-α (light grey columns) and TGF-β1 (dark grey columns), respectively, from the above experiment in arbitrary units. Each value was normalized to the respective GAPDH mRNA expression. Shown are the means of six independent experiments. The standard errors of the mean (SE) are shown as error-bars. Asterisks indicate statistically significant (two-tailed t-test, p ≤ 0.05) alterations compared to the untreated control (white columns).

The data presented above indicate that there is a large variety in the MMP expression patterns between different cell-lines and primary cells and that these expression patterns even change with the duration of cell culture. Therefore, we tested whether GBM cell-lines also may be different in their ability to regulate MMP expression after stimulation with cytokines like TNF-α or TGF-β1. U251 and U373 GBM cells were successively adjusted to grow in medium without FCS to avoid artefacts by starvation shock and then stimulated either with TNF-α (10 ng/ml) or TGF-β1 (10 ng/ml). Expression of MMP-1, -9, -11, -19 and MMP-24 was determined by semiquantitative RT-PCR (Figure 4). In both cell-lines TNF-α stimulated expression of MMP-9 and MMP-19 (Figure 4). MMP-1 mRNA expression was significantly increased in U373 cells by TNF-α stimulation, whereas MMP-1 expression in U251 cells remained unaffected (Figure 4). TGF-β1 had no impact on MMP expression in U251 cells. However, in U373 cells MMP-11 and MMP-24 expression was significantly increased (Figure 4).

Together these data suggest that not only is the expression pattern of MMPs different in the analysed GBM cell-lines and primary cells, but that also regulation of MMP expression by cytokines diverges.

## Discussion

Active migration of cells and invasion of the tumor into the surrounding normal brain tissue are key features of glioblastomas [2,3]. It has been shown that MMP-9, MMP-2 and its activator MMP-14 are involved in these processes [37-40]. Therefore, these MMPs have been extensively studied in several glioblastoma cell-lines (Table 1 and references stated there) and first clinical trials are performed for treatment of GBM patients by inhibition of MMPs [41]. However, data about expression of other MMPs in these cells are limited (Table 1). The only comprehensive study is an analysis of U251 cells by quantitative real time PCR for expression of all known 23 human MMPs [31].

Since it has been reported that there are differences in the expression patterns of MMPs in different cell-lines, even if they originate from the same type of tissue [42], we analysed 7 glioblastoma cell-lines (SNB-19, GaMG, U251, U87, U373, U343 and U138) for expression of 15 MMPs (MMP-1, -8, -9, -10, -11, -13, -17, -19, -20, -21, -23, -24, -26, -27, -28) by semiquantitative RT-PCR. For expression of the remaining MMPs in most of the analyzed cell-lines there are already data available in the literature. These literature-data and our own results are summarized in Table 1.

It is conspicuous that MMP-13, -17, -19 and -24 were expressed by all analyzed cell-lines, whereas MMP-20 and MMP-21 were not expressed by any of the GBM cells (Table 1, Figure 1, Figure 2). We also did not detect any expression of MMP-8, -26 and -27 in any of the cells examined, whereas Nuttall et al. reported detection of mRNA of these MMPs in U251 cells by quantitative real time PCR [31]. This discrepancy may be due to a higher sensitivity of real time PCR in comparison to our semiquantitative approach. However, our results for MMP-8 and MMP-26 in U251 cells match to those obtained by other groups [26,27]. Chernov et al. performed genome-wide transcripitional profiling and quantitative reverse transcription-PCR of U251 cells [26]. Whereas they found expression of MMP-2, -7, -10, -13, -15, and -16, they did not detect MMP-8 expression [26] and Deng et al. were choosing U251 cells for their MMP-26 overexpression experiments, because these cells did not originally display MMP-26 expression [27].

Controversial data about MMP expression have also been reported for MMP-2 in U251, MMP-3 in U87, U373 and U138, MMP-7 in U87 and U138, MMP-9 in SNB-19, U251, U87 and U373, MMP-11 in U251, MMP-12 in U251 and U138, MMP-14 and -15 in U87 and U373 and MMP-16 in U251 cells (Table 1). For detection of these MMPs such diverse methods as semiquantitative RT-PCR [19,27,36], quantitative real time-PCR [26,31,34], gelatin zymography [10,19,20,25,29,30,32,33,36], Northern-blotting [10,15,20,29,32], Western-blotting [10,20,29,30,32,34,36], ^125^I Western-blotting after protein concentration [33] and RNase protection assay [28] have been used. This list implies that different methods may come to dissimilar conclusions, due to disparate sensitivities and due to comparing mRNA expression with protein expression or protein activity. However, in most cases these studies coincide in their results, as can be seen in Table 1 for expression of most MMPs in U251, U87 and U373 cells, suggesting that divergent data may also be caused by other factors. Indeed, we saw fluctuations in MMP-1, -9, -11, -17, -19 and MMP-24 mRNA expression with increasing passages in some GBM cell-lines (Figure 1; Figure 2).

MMPs are highly regulated on the transcriptional and also the protein level [35,38,39] and variations in MMP expression, dependent on the number of passages in cell cultures, have been reported [43]. It was suggested that these variations in MMP expression may be due to *in vitro *selection processes or karyotype evolution where the transcription of either the enzyme and/or its inhibitor may be affected and thus lead to an imbalance in the MMP-regulatory network [43]. However, alterations of MMP expression may also depend on the cells environment. MMP-2, -9 and MMP-14 are differentially upregulated by increasing cellular density [44]. MMP-14 expression also was enhanced if U87 cells were cultured as neurospheres instead as monolayers [45]. Compared to its expression in U251 cells growing in two-dimensional matrix, MMP-12 expression increased during growth in a three-dimensional Tenascin-C matrix [46]. U87 cells displayed low MMP-7 expression in culture, which was much higher after implantation of the cells within the brain of RAG 2/γc immune-deficient mice [47], suggesting that the astrocyte environment may influence MMP expression. Astrocytes in culture produce significant amounts of pro-MMP-2, but undetectable levels of active MMP-2. Co-cultured U251 cells are then able to convert pro-MMP-2 into its active form [48]. Therefore, we wondered if primary cells derived from tumor biopsies from patients will change their MMP expression pattern (Figure 3). We analysed tumor samples of GBM-patients by semiquantitative RT-PCR [16] (and unpublished results). From four of these tumors we isolated and cultured primary cells and analysed them at passage 1, passage 5 and passage 10 (Figure 3). Already passage 1 showed a completely altered MMP expression pattern as seen in the corresponding tumor tissue and this pattern was not stable, but changed with further passages (Figure 3). The pattern was similar to the one seen in established GBM cell-lines, although there were also differences. MMP-1, -11, -17, -23 and MMP-24 expression was stronger in the primary cells. MMP-9 expression showed more alterations during passages in primary cells, but was more stably expressed in the cell-lines. MMP-13 and MMP-28 expression was nearly absent in primary cells, whereas it clearly was visible in the cell-lines (Figure 1).

Together these data indicate that there is a large variety in the MMP expression patterns between different cell-lines and primary cells and that these expression patterns are changeable with duration of cell culture and are highly dependent of cell culture conditions and cell-density. Most importantly, these expression patterns do not match to those seen in GBM tumor tissue of patients.

*In vivo *MMPs are regulated by the surrounding tissue and by growth-factors or cytokines [35,38,39]. Glioblastomas are highly hypoxic and hypoxia upregulates MMP-2 mRNA expression in U87, U251, U373 and LN18 glioblastoma cell-lines by activation of the HIF-1 transcription factor, thereby enhancing their invasive potential [49]. Migration and invasion of U87 and T98G GBM cells is also facilitated by NO, which can be found in high concentrations in glioblastoma tissue [50]. NO stimulates MMP-1 expression and activity [50]. Epidermal growth factor (EGF) raises MMP-14 expression in U251 cells, but does not influence MMP-15, -16 or MMP-24 [34]. MMP-2 expression and secretion is induced by IL-6 in U87 cells [51]. However, IL-6 action seems to be cell-line specific, since U343 cells were not affected [51]. We analysed the effect of tumor necrosis factor-α (TNF-α) and transforming growth factor-β1 (TGF-β1) on MMP-1, -9, -11, -19 and MMP-24 expression in U251 and U373 cells (Figure 4). TNF-α and TGF-β1 are inflammatory and immunsuppressive cytokines, respectively. They have been implicated in migration and invasion of glioma cells *in vitro *[52-54]. However, it seems that they have converse impact [52]. In U251 and in U373 cells TNF-α stimulated expression of MMP-9 and MMP-19 (Figure 4). MMP-1 mRNA expression was significantly increased in U373 cells by this cytokine, whereas its expression in U251 cells remained unaffected (Figure 4). This may be due to the high basal level of MMP-1 expression displayed by U251 cells, which does not allow a further increase, or it may be a cell-line specific effect. Such an effect has been observed for MMP-1, -2, -3 and MMP-7 regulation by TNF-α and TGF-β1, which caused marked induction of expression only in some GBM cell-lines, but not in others [15]. TNF-α enhances invasivenes of T98G cells through MMP-3 induction, but has no effect on MMP-1, -2 or MMP-9 expression [55]. However, in U251 cells TNF-α inhibits MMP-2 expression and decreases invasiveness through an extracellular matrix [56]. In A172 cells TNF-α induces gene expression and protein secretion of MMP-9 [57]. TGF-β1 alone had no effect on MMP-9 production. However, when it was added together with TNF-α a significant dose-dependent inhibition of MMP-9 secretion was observed [57]. TGF-β1 displayed inconsistent effects on adhesion and invasiveness, depending on the cell-line examined. The invasive potential of U138 cells was markedly reduced whereas U373 cell invasion remained unchanged [58]. TGF-β1 caused significant induction of MMP-11 and MMP-24 expression in U373 cells, whereas we did not find any impact on MMP expression in U251 cells (Figure 4). In U87 and LN229 cells TGF-β1 upregulates MMP-2 [53,54]. Thus, the transcriptional modulation of MMP genes in response to TNF-α or TGF-β1 is not consistent, but highly cell-line specific [15].

## Conclusions

Data from literature and our own results suggest that not only is the expression pattern of MMPs highly variable, dependent on the cell-line and the culture-conditions used, but also regulation of MMP expression by cytokines diverges. This is of high impact, if results from cell-culture experiments will be brought forward to the situation in tumor tissue or even will be commuted to clinical applications.

## Methods

### Cell-lines and cell culture

Expression of MMPs was investigated in seven human GBM cell-lines and in four GBM primary cell cultures, which were prepared from GBM tissue samples of patients as described previously [19]. SNB-19, U251, U87, U373, U343 and U138 were originally purchased from ATCC (American Type Culture Collection, Rockville, MD) [23]. GaMG cells were established from a patient by the Gade Institute of the University Bergen, Norway [59].

Primary cells and cell-lines were cultured in Dulbecco's modified Eagle's medium (DMEM) (CytoGen, Sinn, Germany) supplemented with 10% fetal bovine serum (FCS), 4× nonessential amino acids, 3 mM L-Glutamine, Penicillin (100 U/ml) and Streptomycin (100 mg/ml) (all from Invitrogen, Carlsbad, USA). Cells were grown as monolayer in 75 cm^2 ^flasks (Corning, New York, USA) at 37°C in a humidified atmosphere containing 5% CO_2_. Primary cells were grown from passage number 1 to 10 and cell-lines were passaged 15 times (starting passage was determined as passage 0).

### Stimulation of cells with TNF-α and TGF-β1

The FCS concentration in the culture medium of U251 and U373 cells was successively reduced from 10% via 5% and 2.5% to 0% to adjust the cells to grow in medium without FCS. Once cells reached 70% confluency human recombinant TNF-α (10 ng/ml) or TGF-β1 (10 ng/ml) (both from Sigma-Aldrich, St. Louis, USA) was added for 48 h [60]. Control cells were treated with PBS containing 2 mg/ml BSA.

### RNA extraction and semiquantitative RT-PCR

Total RNA was isolated from the GBM cells using the Nucleo-Spin RNA/Protein Kit (Macherey-Nagel, Düren, Germany) following the manufacturer's protocol. During RNA extraction contaminating genomic DNA was digested with the provided DNase I, as suggested by the manufacturer. Total RNA was eluted in a maximum volume of 60 μl RNase-free water and purified samples stored at -80°C.

The mRNA expression level of MMPs was evaluated by semiquantitative RT-PCR. Total RNA (1-5 μg) was reverse-transcribed using the RevertAid H minus first strand cDNA synthesis kit (Fermentas, Ontario, Canada) and the provided oligo(dT)_18 _primer. First-strand cDNA synthesis was carried out at 42°C for 60 min in a final reaction volume of 20 μl and synthesized cDNA stored at -20°C. The amount of cDNA was normalized to the intensity of the PCR product of the ubiquitously expressed gene glyceraldehyde-3-phosphate dehydrogenase (GAPDH) which was used as internal control [61]. Polymerase chain reactions were carried out on the Thermocycler T3 (Biometra, Göttingen, Germany). All primer sequences and PCR conditions are listed in Table 2. Template DNA was mixed with 2.5 U Taq polymerase, 10× Buffer with 1.5 mM MgCl_2 _(Eppendorf, Hamburg, Germany), 200 μM dNTPs (Fermentas, St. Leon Rot, Germany), 0.4 μM of both, forward and reverse primers (Table 2) and formamide (used optionally at a final concentration of 4%) in a total reaction volume of 25 μl. PCR was performed as follows: 5 min at 94°C; 21-32 cycles (Table 2) of 30 sec at 94°C, 30 sec at the optimised annealing temperature (Table 2), 30 sec at 72°C; followed by a termination step of 10 min at 72°C. PCR products were separated on 1% agarose gels (Sigma-Aldrich, Steinheim, Germany) containing 0.07 μg/ml ethidium-bromide (Roth, Karlsruhe, Germany).

**Table 2:** Primers and conditions used for semiquantitative RT-PCR screening

Gene	Forward primer	Reverse primer	Tm (°C)	Cycles	cDNA fragment size (bp)	Genomic DNA (bp)
MMP-1	5'-AAGGCCAGTATGCACAGCTT-3'	5'-TGCTTGACCCTCAGAGACCT-3'	57	32	480	1.060
MMP-8	5'-TCTGCAAGGTTATCCCAAGG-3'	5'- ACCTGGCTCCATGAATTGTC-3'	57	32	154	852
MMP-9	5'-CCTGCCAGTTTCCATTCATC-3'	5'-GCCATTCACGTCGTCCTTAT-3'	58	32	455	957
MMP-10	5'-CCAGTCTGCTCTGCCTATCC-3'	5'- CATCTCAGATCCCGAAGGAA-3'	55	32	819	4.199
MMP-11	5'-GGGGATGTCCACTTCGACTA-3'	5'-CAGTGGGTAGCGAAAGGTGT-3'	50	32	165	308
MMP-13	5'-AACATCCAAAAACGCCAGAC-3'	5'-GGAAGTTCTGGCCAAAATGA-3'	57	32	166	1.117
MMP-17	5'-GGAGCTGTCTAAGGCCATCA-3'	5'-CGACAGGTTCCTCTTGTTCC-3'	56	32	190	474
MMP-19	5'-CAGCCTCGTTGTGGCCTAGA-3'	5'-ACCAGCCTGCACCTCTTGGA-3'	55	32	207	430
MMP-20	5'-CGACAATGCTGAGAAGTGGA-3'	5'-ATCTTTGGGGAGGTGGAATC-3'	57	32	169	975
MMP-21	5'-GACGACGACGAGCACTTCAC-3'	5'-TTTCCTGTCTGACCAGTCCA-3'	53	32	180	431
MMP-23	5'-TGGGACCACTTCAACCTCAC-3'	5'-CGTGTTGTGAGTGCATCAGG-3'	55	32	412	938
MMP-24	5'-GAACCTGTGGGCAAGACCTA-3'	5'-TGACAACCAGAAACTGAGCG-3'	52	32	214	2.650
MMP-26	5'-GATATGAAGCCATCCGCAGT-3'	5'-GCTGGAAGGTTCTAGGGTCG-3'	58	32	378	1.470
MMP-27	5'-TTGTTTCTTGTGGCTGCTCA-3'	5'-GCTAAGCCAAAGGAACCCAC-3'	53	32	194	376
MMP-28	5'-CACCTCCACTCGATTCAGCG-3'	5'-AAAGCGTTTCTTACGCCTCA-3'	57	32	208	376
GAPDH	5'-GCAGGGGGGAGCCAAAAGGG-3'	5'-TGCCAGCCCCAGCGTCAAAG-3'	68	21	566	851

Tm: annealing temperature, bp: base pairs.

### Densitometric quantification and statistical analysis

The intensity of ethidium bromide fluorescence was densitometrically analyzed using the BioDocAnalyze software (Biometra, Göttingen, Germany). The DNA bands were normalized to the respective fragment intensity of the housekeeping gene GAPDH. Statistical analysis was performed using Microsoft Office Excel 2003 (Microsoft Deutschland, Unterschleißheim, Germany). Values were expressed as means ± standard error of the mean (SE). Statistical significance was defined by two tailed t-tests and p ≤ 0.05 was considered to be significant.

## Competing interests

The authors declare that they have no competing interests.

## Authors' contributions

CH and JA contributed equally as primary authors of the manuscript. They designed and supervised the study. SH performed the semiquantitative RT-PCRs. DR and BS analysed MMP expression after cytokine stimulation. SH, DR and BS did the statistical analysis and generated figures. RIE and GHV participated in the study design and critically reviewed the manuscript. All authors read and approved the final manuscript.
